# Two chitinase-like proteins abundantly accumulated in latex of mulberry show insecticidal activity

**DOI:** 10.1186/1471-2091-11-6

**Published:** 2010-01-28

**Authors:** Sakihito Kitajima, Kaeko Kamei, Shigeru Taketani, Masamitsu Yamaguchi, Fumiko Kawai, Aino Komatsu, Yoshihiro Inukai

**Affiliations:** 1 Department of Applied Biology, Kyoto Institute of Technology, Matsugasaki Sakyo-ku, Kyoto 606-8585, Japan

## Abstract

**Background:**

Plant latex is the cytoplasm of highly specialized cells known as laticifers, and is thought to have a critical role in defense against herbivorous insects. Proteins abundantly accumulated in latex might therefore be involved in the defense system.

**Results:**

We purified latex abundant protein a and b (LA-a and LA-b) from mulberry (*Morus *sp.) and analyzed their properties. LA-a and LA-b have molecular masses of approximately 50 and 46 kDa, respectively, and are abundant in the soluble fraction of latex. Western blotting analysis suggested that they share sequence similarity with each other. The sequences of LA-a and LA-b, as determined by Edman degradation, showed chitin-binding domains of plant chitinases at the N termini. These proteins showed small but significant chitinase and chitosanase activities. Lectin RCA120 indicated that, unlike common plant chitinases, LA-a and LA-b are glycosylated. LA-a and LA-b showed insecticidal activities when fed to larvae of the model insect *Drosophila melanogaster*.

**Conclusions:**

Our results suggest that the two LA proteins have a crucial role in defense against herbivorous insects, possibly by hydrolyzing their chitin.

## Background

Laticifer cells of plants are unique in shape, development and function. These cells form long tubular or branched structures that run through the plant body, and large amounts of cytoplasm are exuded when the plant body is cut. The cytoplasm is a sticky white fluid called latex, and in the case of the para rubber tree (*Hevea brasiliensis*) and rubber fig (*Ficus elastica*), insoluble latex particles are a well-known commercial source of natural rubber [1]. Laticifers are not present in all plant species. For example, well-studied model plants such as *Arabidopsis*, rice, wheat, and tobacco, do not have laticifers. However, they have been reported in about 12 500 plant species in 22 families, including monocots and dicots, and are estimated to exist in up to 20 000 species in 40 families [1].

One important feature of laticifers is that they contain various toxic compounds in the latex; for example, the neurotransmitter dopamine in the Persian poppy (*Papaver bracteatum*), narcotic alkaloid morphine in the opium poppy (*Papaver somniferum*), and insecticidal compounds such as the glycosidase inhibitors 1,4-dideoxy-1,4-imino-d-arabinitol (d-AB1) and 1-deoxynojirimycin (DNJ) in mulberry [2,3]. In addition, cysteine protease in latex of papaya (*Carica papaya*) and wild fig (*Ficus virgatalatex*) is toxic to caterpillars of herbivorous insects [4]. The compounds are often highly condensed in the latex; for example, d-AB1, DNJ and its related compound 1,4-dideoxy-1,4-imino-D-ribitol are present at 8-18% dry weight in latex of mulberry leaf; 100 times higher than that in the whole leaf [5]. Rubber is also toxic to herbivorous insects because its stickiness limits movement of their mouthparts [6].

These findings suggest that laticifers are specialized cells that have a role in defending plants against herbivorous insects. The distribution of laticifers is associated with vascular tissue, which transports metabolites, water, minerals and signaling molecules through the plant body, and is one of the most important plant tissues. Laticifers might protect the vascular system. Long tubular or branched network structures are effective in this role, because they enable laticifers to exude large amounts of latex at damaged sites. In the present study, we speculate that proteins that are abundant in latex have a defensive role against herbivorous insects. Here, we report that two proteins that are abundant in the soluble fraction of mulberry latex have insecticidal activities against the model insect *Drosophila melanogaster*, possibly through their chitinase activity.

## Results

### Purification of LA-a and LA-b

**Figure 1:**
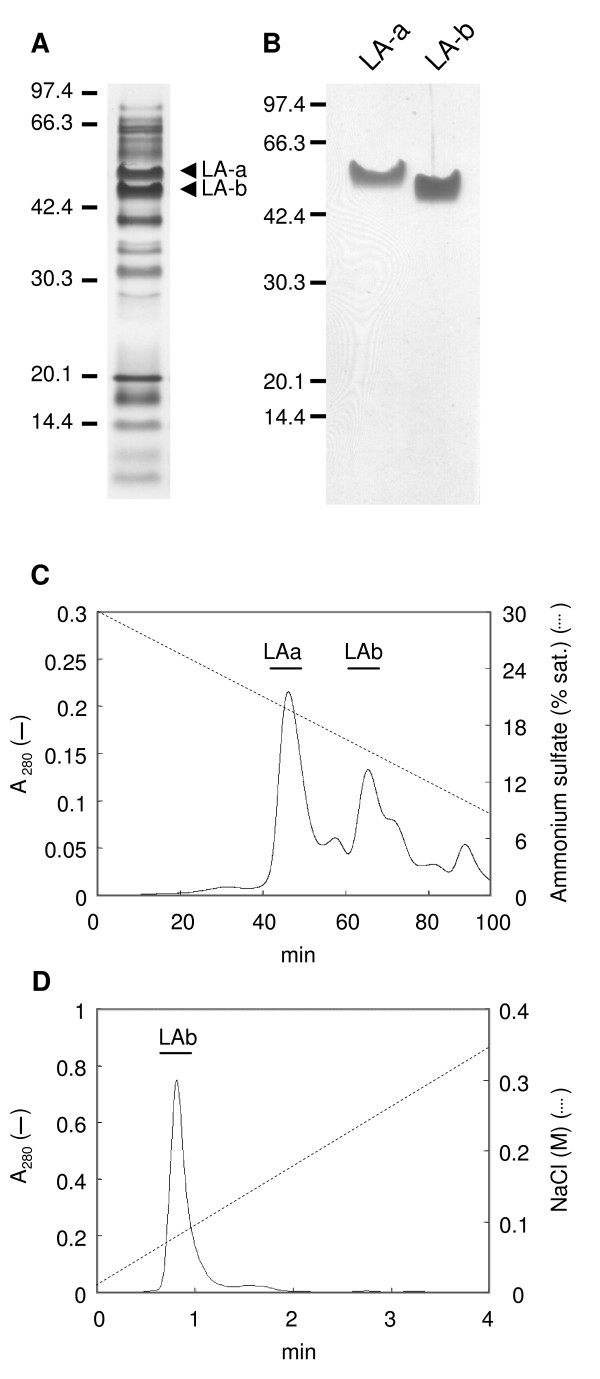
**Purification of LA-a and LA-b**. (A) SDS-PAGE of soluble fraction of mulberry latex obtained from petiole. Soluble proteins obtained from 0.025 μl of latex were loaded. (B) SDS-PAGE of purified LA-a and LA-b. One microgram of protein was loaded per lane. The proteins were stained with silver. (C and D) Hydrophobic interaction chromatography (phenyl sepharose, C) and cation-exchange chromatography (UNOS, D).

SDS-PAGE analysis indicated that two proteins with a molecular mass of approximately 50 and 46 kDa were abundant in the soluble protein fraction of latex obtained from the cut petiole (Figure 1A) of mulberry. Similar SDS-PAGE results were observed for latex samples taken from a young branch (<1 year old) of mulberry (data not shown). Latex is thought to have a crucial role in defense against herbivorous insects, therefore, the two abundant proteins were expected to have insecticidal activity. We named the proteins latex abundant protein a (LA-a; 50 kDa) and latex abundant protein b (LA-b; 46 kDa), and purified them to analyze their properties. Cation-exchange chromatography (CM-cellulofine C-200), followed by hydrophobic interaction chromatography (phenyl sepharose) (Figure 1C) gave a single band of LA-a in SDS-PAGE (Figure 1B). LA-b was purified further by cation-exchange chromatography (UNOS) (Figure 1D) to give a single band (Figure 1B). From 3 ml of latex, we obtained 5.8 and 1.1 mg of purified LA-a and LA-b, respectively.

### Amino acid sequence analysis

**Figure 2:**
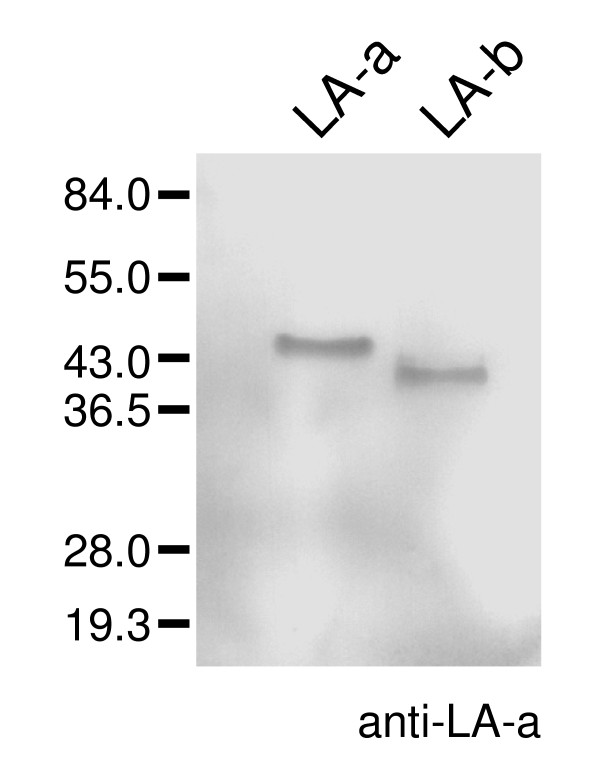
**Cross-reactivity of LA-b to anti-LA-a antibody**. Purified LA-a and LA-b (0.1 μg per lane) were electrophoresed, blotted onto PVDF membrane and immunoreacted with rat polyclonal anti-LA-a antibody.

**Figure 3:**
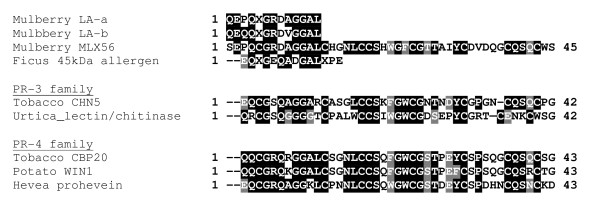
**Comparison of the amino acid sequences of LA-a and LA-b with chitin-binding domains of other plant proteins**. Conserved amino acid residues are shown as white letters on black background and similar residues are highlighted in grey. X in mulberry and *Ficus *proteins indicate residues that could not be identified by protein sequencers. The proteins shown here are LA-a and LA-b from mulberry (this study), MLX56 from mulberry (DNA database EF535852, [7]), 45-kDa allergen from *F. benjamina *[8], hevein-like protein from elderberry (AF074386, [19]), CHN50 from tobacco (X51599, [20]), CBP20 from tobacco (S72424, [21]), WIN1 from potato (X13497), prohevein from *H. brasiliensis *(M36986, [22]).

A polyclonal antibody raised against LA-a also recognized LA-b (Figure 2), which suggests that they share sequence similarity. Edman degradation showed that N-terminal sequences of LA-a and LA-b were similar to residues 22-34 of latex protein MLX56 of mulberry [7] (Figure 3). The calculated molecular mass of MLX56 from residue 22 (N terminus of mature form) to the C terminus is 45.2 kDa. MLX56 has two chitin-binding domains in the N-terminal half and a plant chitinase domain in the C-terminal half. It was thus suggested that LA-a and LA-b have chitinase domains. The N-terminal sequences of LA-a and LA-b were also similar to that of a latex protein from another *Moraceae *plant, *Ficus benjamina *[8] and to the chitin-binding domains, located in the N-terminal end, of plant pathogenesis-related (PR) proteins group 3 (chitinase) and 4 (Figure 3). The molecular masses of PR-3 and PR-4 (20-40 kDa) are much lower than those of LA-a and LA-b.

### Analysis of glycosylation

**Figure 4:**
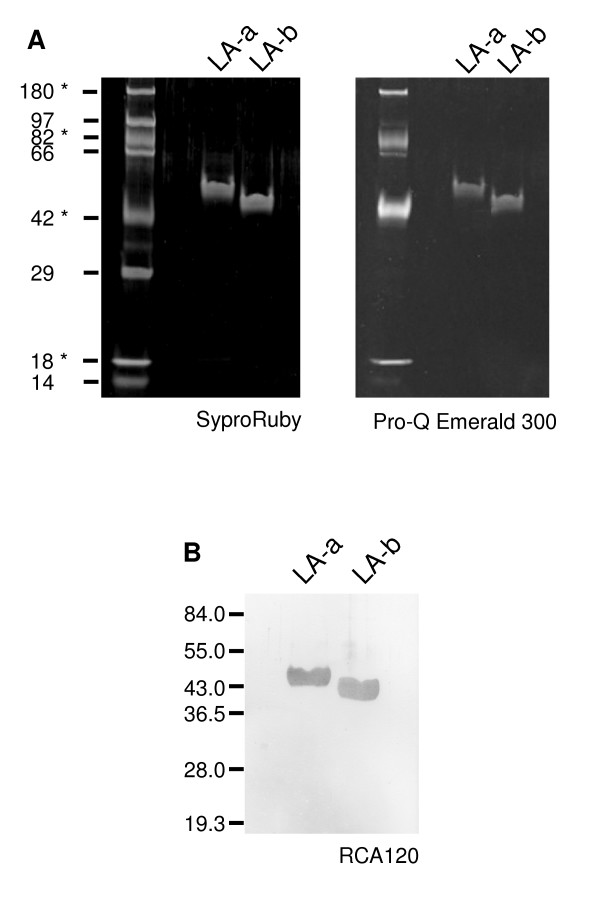
**Analysis of glycosylation of LA-a and LA-b**. (A) Purified LA-a and LA-b (3 μg per lane) were electrophoresed and stained: SyproRuby for detection of proteins, and Pro-Q emerald 300 for detection of glycoproteins. CandyCane glycoprotein molecular weight standards (0.5 μg for each band) were also loaded. Numbers marked with a star indicate glycoproteins. (B) Purified LA-a and LA-b (1 μg per lane) were electrophoresed, blotted onto PVDF membrane and reacted with lectin RCA120.

LA-a and LA-b proteins were stained by Pro-Q Emerald 300 (Figure 4A), which stains glycoproteins, and indicates that they are glycosylated. To study the glycosylation of LA-a and LA-b in detail, we tested 15 lectins (ConA, DBA, LCA, PHA-E4, PNA, RCA120, UEQ-I, WGA, ABA, DSA, Lotus, MAM, PHA-L4, SBA, and SSA; data not shown). Of these, RCA120, which is specific for galactose, reacted strongly and reproducibly with LA-a and LA-b (Figure 4B). This is a unique feature because common chitinases of plants are not glycosylated.

### Analysis of chitinase and chitosanase activities

**Figure 5:**
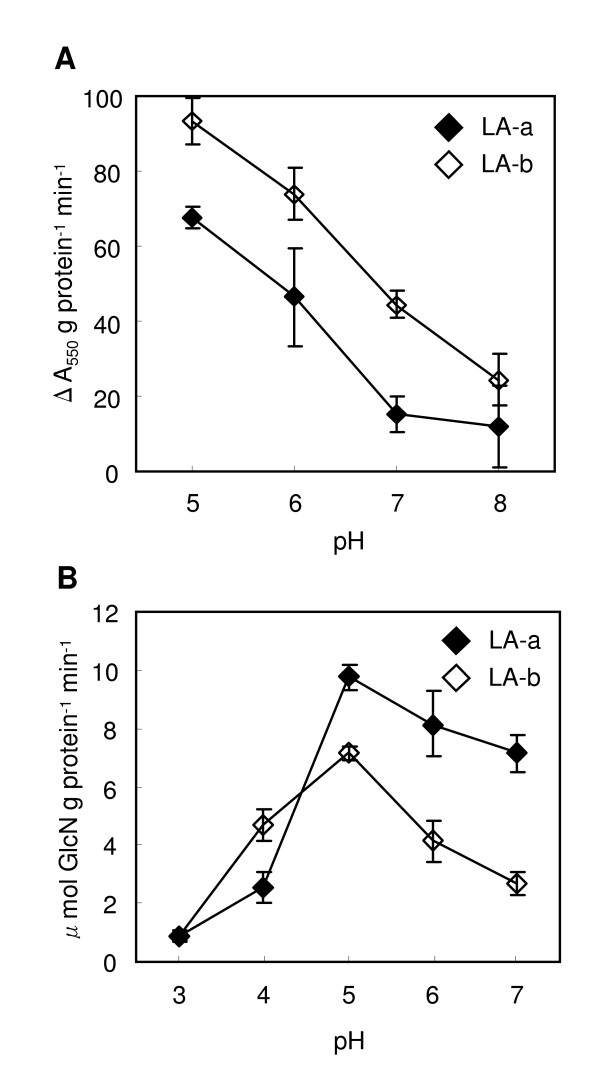
**Chitinase and chitosanase activities of LA-a and LA-b under various pH conditions**. (A) Colorimetric chitinase assay using CM-chitin-RBV as a substrate. Increase in hydrolyzed chitin was monitored by absorbance at 550 nm. (B) Fluorometric chitosanase assay using soluble chitosan as a substrate. Reaction product glucosamine (GlcN) was labeled with fluorescamine and monitored fluorometrically (excitation: 395 nm; emission: 493 nm). Graph shows average ± SD (n = 5).

The sequence similarity between the chitin-binding domains of known chitinases and the N-terminal sequences of LA-a and LA-b suggests that they are also chitinases. We therefore examined the enzyme activities of LA-a and LA-b. When using carboxymethylated (CM) chitin labeled with remazol brilliant violet (RBV) as a substrate, LA-a and LA-b showed chitinase activity (Figure 5A). When activity was tested at a pH range of 5-8, maximum activity was observed at pH 5. We could not test the activity at lower pH because the substrate was precipitated under these conditions. Mayer *et al*. [9] have compared CM-chitin-RBV-degrading activity of 7 chitinase isoforms of orange (*Citrus sinensis*) with that of hen egg lysozyme. In that study, the activity of 2 isoforms was 100 times higher than that of hen egg lysozyme, and the activity of 5 isoforms was 2.5-10 times higher. We also compared the activity of LA-a and LA-b with that of hen egg lysozyme. At pH 5, chitinase activity of LA-a and LA-b was 1/10 and 1/7, respectively, of that of hen egg lysozyme at pH 5 (data not shown). Compared to the orange chitinases, LA-a and LA-b thus had markedly lower chitinase activity.

LA-a and LA-b also were able to hydrolyze chitosan, which is partly deacetylated and therefore solubilized chitin. Chitosanase activity was tested at a pH range of 3-7, and maximum activity was observed at pH 5 (Figure 5B). At pH 5, the chitosanase activity of LA-a and LA-b was 1.8 and 1.4 times higher, respectively, than that of hen egg lysozyme (data not shown). In the report by Mayer *et al*. [9], chitosanase activity of 2 chitinase isoforms was 30 and 100 times higher than that of hen egg lysozyme, and the activity of 4 isoforms was 1.7-3.5 times higher. One isoform showed no chitosanase activity.

These results indicated that LA-a and LA-b had chitinase and chitosanase activities, although the activities were relatively low compared to those of orange chitinases.

### Insecticidal activity against larvae of *D. melanogaster*

**Figure 6:**
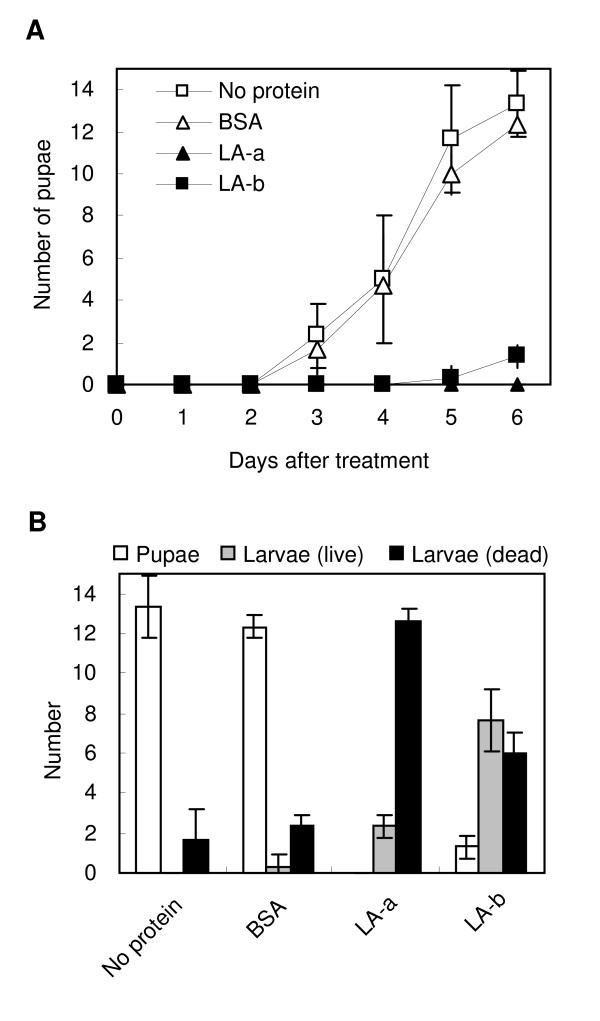
**Insecticidal activity of LA-a and LA-b against *D. melanogaster***. Fifteen 2-day-old larvae (second instar) per vial were fed with an artificial diet that contained 0.1% (w/w) LA-a, LA-b, BSA or no protein. (A) Numbers of pupae developed in 6 days. (B) Numbers of pupae and live and dead larvae 6 days after feeding. Results indicate average ± SD of 3 vials.

Finally, we examined insecticidal activity of LA-a and LA-b using larvae of the model insect *D. melanogaster*. *D. melanogaster *has some advantages over other insects; it is easy to rear, and its small size and short life cycle reduces food requirements for the assay. In addition, because *D. melanogaster *does not eat mulberry, it was not expected to have any existing mechanism for overcoming the potential toxicity of mulberry protein. When 2-day-old larvae (second instar) were fed an artificial diet that contained 0.1% (w/w) LA-a or LA-b, very few developed into pupae over 6 days of incubation (Figure 6A). After 6 days, 80% of larvae fed with LA-a and 40% of those fed with LA-b were found to be dead (Figure 6B). In contrast, when fed with diets that contained bovine serum albumin or no protein instead of LA-a or LA-b, almost all larvae developed into pupae (Figure 6A). This result indicated that LA-a and LA-b had insecticidal activity against larvae of *D. melanogaster*.

## Discussion

In this study, we showed that the two proteins LA-a and LA-b were abundant in the soluble fraction of mulberry latex, and had low, but significant chitinase and chitosanase activities. In addition, both LA proteins showed insecticidal activity against larvae of *D. melanogaster*. The finding that these major proteins have insecticidal activity supports the idea that laticifers are specialized cells for defense against herbivorous insects.

In a recently published study [7] on mulberry latex protein MLX56, it has been shown that MLX56 is glycosylated and has growth-inhibiting (not insecticidal) activity on the lepidopteran caterpillars of cabbage armyworm (*Mamestra brassicae*) and Eri silkworm (*Samia richini*), but not on mulberry specialist silkworm (*Bombyx mori*). Similarities between LA-a and LA-b proteins and MLX56, with respect to N-terminal amino acid sequence and electrophoretic mobility in SDS-PAGE, suggest that they are the same or similar proteins. However, contrary to our results, MLX56 has been reported to have no chitinase activity [7]. Enzyme activity of MLX56 may need to be analyzed using various substrates. In our study, we observed chitinase activity of LA-a and LA-b when using CM-chitin-RBV and chitosanase activity when using chitosan (Figure 5), but the activity was hardly detectable when 4-methylumberryferyl β-D-N, N', N"-triacetylchitotriose (4-MU-(GlcNAc)_3_), a common substrate for plant chitinase assay, was used (data not shown). Substrate specificity of LA proteins and MLX56 could be different from that of common plant chitinases.

Chitin, a substrate of chitinase, forms the cuticle of the exoskeleton in insects, and should be the target of LA-a and LA-b in the larvae of *D. melanogaster *and other insects. In addition to external body surfaces, the inside of the mouthpart is also a possible target of LA-a and LA-b because the pH inside the mouthpart is expected to be near that of crude extract of mulberry tissues (weekly acidic or neutral) and to be optimal for the chitinase activity of LA proteins.

Plant chitinase is well known to have antifungal activity, but only a few studies have investigated its toxicity to insects. It has been reported that transgenic tomato that accumulated poplar (*Populas *sp.) chitinase inhibited development of Colorado potato beetle (*Leptinotarsa decemlineata) *larvae, and the amount of chitinase in the leaves was >0.3% (w/w) [10]. Two papers have reported that insect chitinase showed toxicity to insects; chitinase of tobacco hornworm (*Manduca sexta*) included at 2% in an artificial diet showed 100% mortality of larvae of merchant grain beetles (*Oryzaephilis mercator*) [11], and transgenic tobacco leaves that accumulated chitinase of tobacco hornworm at 0.02-0.03% of total leaf protein inhibited growth of tobacco budworm (*Heliothis virescens*) larvae [12].

The insecticidal activity of LA proteins might not be caused simply by chitinase activity. It could be that a domain of LA proteins with unidentified biochemical activity cooperates with the chitinase domain to enhance insecticidal activity. Estimated molecular masses of LA-a and LA-b (50 and 46 kDa) were higher than those of common plant chitinases (20-40 kDa). This is at least partly caused by glycosylation, but it also suggests that the proteins might have another functional domain and biochemical function, working in cooperation with chitinase activity for efficient toxicity. Further analysis is needed to understand the molecular mechanism of insecticidal activity of LA-a and LA-b.

Unlike some other insects that eat leaves of latex-producing plants, silkworm eats mulberry leaves without removing the latex by cutting or trenching veins (see [13,14] for reviews). Therefore, silkworms probably have a mechanism for detoxifying the insecticidal activity of LA proteins, and are unsuitable for studying potential insecticidal activity of LA proteins. One of the reasons why we used *D. melanogaster *in this study is that this insect does not eat mulberry and thus is not expected to overcome potential toxicity of LA proteins. It would be interesting to know to which species of insect other than *D. melanogaster *(*e.g*. pest insects of economically important corps) the LA proteins are toxic. Some insect species including silkworm would be tolerant to LA proteins, and it would be interesting to investigate the mechanism by which they can overcome the toxicity of the LA proteins.

## Conclusions

In the present study, we indicated that two chitinase-like proteins (LA-a and LA-b) were abundantly accumulated in latex of mulberry and suggested that they have a crucial role in defense against herbivorous insects, possibly by hydrolyzing their chitin.

## Methods

### Plant material

Mulberry (*Morus alba *L. cv. Minamisakari) was maintained at the Center for Bioresource Field Science, Kyoto Institute of Technology.

### Purification of LA proteins

Latex exuded from the cut petiole of mulberry was mixed rapidly with an equal volume of buffer A (100 mM potassium phosphate, 10 mM EDTA, pH 6.7) supplemented with 0.1% (v/v) β-mercaptoethanol, frozen in liquid nitrogen, and stored at -80°C. Six milliliters of the latex/buffer A mixture was diluted 4 times with buffer A and centrifuged at 18 000 *g *for 30 min. The supernatant was fractionated by ammonium sulfate between 30 and 80% saturation. Precipitated protein was dissolved in 4 ml of buffer B (10 mM potassium phosphate, 1 mM EDTA, pH 6.0) and dialyzed against the same buffer overnight, using a dialysis membrane with a molecular weight cut-off of 25 kDa (Spectra/Por 7; Spectrum Laboratories Inc., Rancho Dominguez, CA, USA). The sample was diluted to 50 ml and loaded onto a CM-Cellulofine C-200 (Seikagaku Kogyo KK, Tokyo, Japan) cation-exchange column (26 mm i.d. × 100 mm) equilibrated with buffer B. Protein fractions that contained LA-a and LA-b were eluted with buffer B that contained 0.2 M KCl at a flow rate of 1.0 ml min^-1^. Eluted protein solution was brought to 30% saturation with ammonium sulfate, and hydrophobic interaction chromatography was carried out on a HiLoad 16/10 phenyl sepharose HP column (16 mm i.d. × 100 mm; GE healthcare, Piscataway, NJ, USA) equilibrated with buffer B that contained ammonium sulfate at 30% saturation. The column was developed with a linear gradient of 30-0% saturation of ammonium sulfate at a flow rate of 2 ml min^-1^. Fractions that contained LA-b were desalted twice by gel filtration chromatography using PD10 (GE healthcare) and 10DG (Bio-Rad, Hercules, CA, USA) column, and further purified on a UNOS1 cation-exchange column (Bio-Rad) eluted with a linear gradient of 0-0.5 M NaCl in buffer B at a flow rate of 3.0 ml min^-1^. Purified LA-a and LA-b proteins were concentrated with Microcon YM-10 (Millipore Corp., Bedford, MA, USA) to 1.0 mg ml^-1^, frozen with liquid nitrogen, and stored at -80°C. All purification steps were performed at 4°C. All chromatography steps were carried out using a BioLogic Duo-Flow chromatography system (Bio-Rad). Protein concentrations were determined according to the equation: protein concentration (mg ml^-1^) = 1.45 A_280 _- 0.74 A_260 _[15].

### Western blotting

A rat polyclonal antibody was raised against purified LA-a by a standard procedure. For western blotting, protein samples were separated by SDS-PAGE and electroblotted onto polyvinylidene difluoride (PVDF) membrane using the buffer system of Kyhse-Anderson [16]. The membrane was reacted with the polyclonal anti-LA-a antibody, followed by alkaline phosphatase-conjugated anti-rat antibody according to Coligan *et al*. [17]. A mixture of 5-bromo-4-chloro-3-indolyl phosphate (BCIP) and nitro blue tetrazolium (NBT) (Promega, WI, USA) was used for color development.

### Edman degradation

Cysteine residues of proteins were alkylated by treatment with iodoacetamide according to Coligan et al. [17]. Proteins were then purified on a C18 reversed-phase column (4.6 × 150 mm; TSK gel ODS-100S; Toso, Tokyo, Japan) eluted with a linear gradient of 5-50% acetonitrile in 0.1% trifluoroacetic acid at a flow rate of 1.0 ml min^-1^. Eluted proteins were concentrated by evaporation and analyzed by automated protein sequencer PPSQ-21A (Shimadzu, Japan).

### Analysis of glycosylation

Protein samples were separated by SDS-PAGE and the gel was stained, first with Pro-Q Emerald 300 to detect glycosylated proteins, and then with SYPRO Ruby stain (both from Invitrogen, Carlsbad, CA, USA) for total proteins, according to the manufacturer's instructions. As a control, CandyCane glycoprotein molecular weight standards (Invitrogen) were used. For the lectin blot, protein samples were separated by SDS-PAGE and electroblotted onto a PVDF membrane as described above. The membrane was reacted with biotinylated RCA120 (Vector Laboratories, Burlingame, CA, USA), and subsequently with alkaline phosphatase-conjugated streptavidin. A mixture of BCIP and NBT was used for color development. Fifteen other lectins (ConA, DBA, LCA, PHA-E4, PNA, RCA120, UEQ-I, WGA, ABA, DSA, Lotus, MAM, PHA-L4, SBA, and SSA) were purchased from Seikagaku Kogyo (Tokyo, Japan).

### Chitinase and chitosanase assay

Colorimetric chitinase assay was performed using CM-chitin-RBV (Loewe Biochemica, München, Germany) as a substrate, according to Mayer *et al*. [9], except that the enzyme reaction was prolonged from 10 min to 2 h. Buffers used were 0.1 M citrate-Na_2_HPO_4 _buffers (pH 5-8). Hydrolyzed chitin was recovered and measured by absorbance at 550 nm. Activity was expressed as Δ550 nm g protein^-1 ^min^-1^. Fluorometric chitosanase assay was performed using soluble chitosan (Chitosan, low molecular weight; Sigma-Aldrich, MO, USA) as a substrate according to Osswald et al. [18], except that the enzyme reaction was prolonged from 30 min to 2 h. Buffers used were 0.1 M citrate-Na_2_HPO_4 _buffers (pH 3-7). Controls consisted of the complete reaction mixtures stopped immediatedly after addition of enzyme, as described by Osswald *et al*. [18]. The reaction product glucosamine (GlcN) was recovered, labeled with fluorescamine (Fluka, Buchs, Switzerland) and monitored fluorometrically (excitation: 395 nm; emission: 493 nm). Chitosanase activity was calculated on the basis of GlcN equivalents using a GlcN standard curve generated under the same conditions as these used for the assays.

### Insecticidal assay

*Drosophila melanogaster *(Canton-S strain) was reared at 25°C on standard yeast agar medium. To test insecticidal activity of LA-a and LA-b proteins, 15 2-day-old larvae (second instar) were placed in a vial that contained 200 mg Formula 4-24 instant *Drosophila *medium (Carolina Biological Supply, Burlington NC, USA), rehydrated with 0.8 ml PBS supplemented with 1 mg LA protein and 10 mg Bacto Yeast Extract (Difco, Detroit, MI, USA) as a nitrogen source. This accounted for 0.1% (w/w) LA protein. For control experiments, the diet was supplemented with the same amount of bovine serum albumin (BSA) or no protein instead of LA protein. The vials were incubated at 25°C.

## Authors' contributions

KK helped with Edman degradation of LA proteins. ST helped with preparation of antibody. MY helped with the *D. melanogaster *experiment. FK, AK and YI carried out all purification and biochemical analysis. SK designed the experiments and wrote the manuscript. All authors read and approved the final manuscript.
